# Integrase inhibitor–based antiretroviral therapy drives gut microbiota remodeling and immune recovery in HIV infection

**DOI:** 10.3389/fmed.2026.1814926

**Published:** 2026-05-12

**Authors:** Banu Karaca, Alper Şener, Figen Kaptan, Deniz Ece, Bahar Örmen, Ayse Caner

**Affiliations:** 1 Department of Infectious Diseases, Atatürk Training and Research Hospital, Katip Çelebi University, Izmir, Türkiye; 2 Department of Basic Oncology, Institute of Health Sciences, Ege University, Izmir, Türkiye; 3 Department of Parasitology, Faculty of Medicine, Ege University, Izmir, Türkiye; 4 Department of Pharmaceutical Microbiology, Faculty of Pharmacy, Ege University, Izmir, Türkiye

**Keywords:** 16S rRNA sequencing, art, CD4^+^ T cells, gut microbiota, HIV infection, integrase inhibitor

## Abstract

**Background:**

The gastrointestinal tract serves as a major viral reservoir in HIV infection, and persistent gut dysbiosis contributes to systemic inflammation and immune dysfunction despite effective antiretroviral therapy (ART). Integrase strand transfer inhibitor (INSTI)–based regimens are now preferred for their efficacy and tolerability; however, their impact on gut microbial restoration remains underexplored.

**Methods:**

This study analyzed fecal microbiota composition in 30 HIV-positive adults—10 ART-naïve, 7 receiving INSTI-based ART for <2 years, and 13 receiving INSTI-based ART for 2–5 years. 16S rRNA gene sequencing of stool samples (V3–V4 regions) was performed to evaluate bacterial diversity and taxonomic shifts. Microbial composition was compared across groups and correlated with CD4^+^ T cell counts.

**Results:**

Alpha diversity showed a non-significant yet progressive increase from ART-naïve to long-term INSTI-treated patients, suggesting partial restoration of microbial diversity. At the phylum level, Firmicutes increased and Bacteroidetes decreased in treated groups, indicating a shift toward eubiosis. Long-term INSTI therapy enriched beneficial taxa such as *Faecalibacterium prausnitzii, Lactobacillus ruminis, Eubacterium biforme,* and *Bifidobacterium longum*, while reducing pro-inflammatory *Bacteroides fragilis* and *Haemophilus parainfluenzae*. Moreover, higher CD4^+^ T cell counts showed a positive correlation with the abundance of short-chain fatty acid–producing bacteria and with an increased Firmicutes/Bacteroidetes ratio, indicating improved mucosal integrity and immune homeostasis.

**Conclusion:**

Integrase inhibitor–based ART promotes partial normalization of gut microbiota composition in HIV-infected patients, enhancing beneficial taxa linked to immune recovery. These findings underscore the potential of microbiota-targeted adjunctive interventions—such as probiotics or dietary modulation—to support mucosal immunity and long-term health in people living with HIV.

## 1 Introduction

Antiretroviral therapy (ART) in HIV infection suppresses viral load and prolongs life expectancy. HIV infection targets CD4^+^ T cells, leading to severe immunosuppression. Approximately 60% of the body’s CD4^+^ T cells reside within the gastrointestinal lymphoid tissues, which serve as a major viral reservoir during HIV infection. It is well known that viral load levels in the gut are higher than in the blood in HIV cases. The virus depletes CD4^+^ T cells in the gut, causing a reduction in Th17 cells, which are essential for maintaining the integrity of the intestinal epithelial barrier. This disruption allows a greater amount of antigens to enter circulation. Although transient, day-to-day fluctuations in the taxonomic composition and relative abundance of commensal microorganisms represent a physiological property of a stable and resilient gut ecosystem, gut dysbiosis denotes a persistent, non-physiological disruption of microbial community structure and function. This maladaptive shift characterized by reduced microbial diversity, loss of key symbionts, and/or expansion of pathobionts has been mechanistically linked to chronic mucosal and systemic inflammation, thereby contributing to the pathogenesis of a wide spectrum of multifactorial disorders, including infectious, autoimmune, metabolic, and neoplastic diseases (1). In addition, alterations in macrophage function can disrupt the microbiota, contributing to both local and systemic inflammation (2). Despite virological suppression in ART-treated individuals, persistent gut dysbiosis and insufficient immune reconstruction in gut-associated lymphoid tissues contribute to metabolic disorders such as cardiovascular disease, premature aging, cognitive deficits, diabetes, liver dysfunction, fat redistribution, and neurocognitive symptoms associated with chronic immune activation and inflammation (3).

By sequencing the 16S rRNA gene, species-specific variable regions can be identified to study microbiota composition at the species level. In a healthy population study the genera *Ruminococcus* and *Bacterioides* were present in all cases while the genus *Prevotella* was found in 30% percent of the subjects (4). Also in another study using fluorescent *in situ* hybridization method two major bacterial constituents of the fecal microbiota were *Bacteroides* and *Clostridium* species (5). Gut microbiota studies have shown decrease in alpha diversity in HIV-positive population when compared with HIV-negative individuals, and even after ART initiation, the microbiota does not return to its pre-infection baseline (6). Similar to conditions like diabetes mellitus and ulcerative colitis, HIV infection alters gut bacterial diversity and abundance, with an observed increase in *Prevotella* species and a decrease in *Bacteroides* species (7).

Overall, the available literature indicates a heterogeneous effect of antiretroviral therapy (ART) on the gut microbiota: whereas some studies report potential microbiota-modulating benefits associated with ART (8), others demonstrate that ART may independently induce or exacerbate dysbiosis irrespective of HIV infection (9, 10). Consequently, the net impact of ART on gut microbial composition remains insufficiently resolved and warrants further investigation.

Fecal microbiota studies in people living with HIV remain limited worldwide. Most research focuses on differences in gut microbiota between HIV-positive and HIV-negative individuals. These studies claim that alpha diversity decrease in HIV-positive populations (8, 11). Some studies have also reported that ART administration may lead to slight increases in microbial diversity, partial restoration of beneficial commensal taxa, and reductions in inflammation-associated bacterial groups—collectively suggesting a modest yet measurable improvement in gut microbial homeostasis (12). However, data specifically evaluating these effects in the context of integrase inhibitor–based regimens remain limited. Integrase inhibitors are in the first line treatment protocols in guidelines, capable of reducing viral load, well tolerated and have high genetic barrier resulting less resistance profile (1, 13).

Altough there are fecal microbiota studies conducted in ART-treated population other than integrase inhibitor-based regiments (PI, NNRTI and NRTI) (14, 15) there are few studies examining whether integrase inhibitor-based regimens increase bacterial diversity in HIV infection (8, 10, 16, 17). Evidence suggests that INSTI-based regimens exert a more beneficial effect on gut homeostasis, whereas protease inhibitor–based regimens are linked to increased microbial translocation and dysbiosis (17, 18). Therefore, this study aimed to characterize the alterations in fecal microbiota composition among ART-naïve HIV patients and those receiving integrase inhibitor–based ART for varying durations.

## 2 Methods

### 2.1 Study population

This study was designed as a cross-sectional study, with all participants recruited between June 15, 2022 and June 15, 2023 at the Infectious Diseases Clinic of a tertiary university research hospital. The study included HIV-positive adults (>18 years) without diabetes mellitus, inflammatory bowel disease, gastrointestinal disorders, recent antibiotic or probiotic use, or any condition potentially affecting gut microbiota. Patients were categorized into three groups: ART-naive (Group 1, *n* = 10), those receiving integrase inhibitor-containing ART for less than 2 years (Group 2, *n* = 7), and those receiving integrase inhibitor-containing ART for 2–5 years (Group 3, *n* = 13). Patients in the ART-treated groups received one of the following INSTI-containing regimens: Bictegravir + Emtricitabine (FTC) + Tenofovir alafenamid (TAF; *n* = 21), Dolutegravir + Tenofovir disoproxil fumarate (TDF) + Emtricitabine (FTC; *n* = 1), and Dolutegravir/Lamivudine (*n* = 9).

At the time of enrollment, all ART-experienced participants (*n* = 20) were receiving INSTI-based regimens, in accordance with national HIV treatment guidelines. No patients in this cohort were receiving protease inhibitor– or NNRTI-based regimens.

No ART regimen changes occurred during the study period. All patients in Groups 2 and 3 maintained their initial INSTI-based combination therapy throughout follow-up, with no switches between INSTIs, no backbone changes, and no treatment interruptions. All participants provided written informed consent before participating in the study. Patient data, including treatment status and duration, age, gender, body mass index (BMI), infection duration, transmission route, sexual orientation, HBV and HCV co-infection status, comorbidities, medication use, CD4/CD8 ratio, and HIV RNA levels, were collected from electronic medical records.

Clinical data and stool samples were collected at the time of enrollment, and no retrospective samples or pre-existing microbiome data were used. Each participant contributed a single stool sample for 16S rRNA sequencing. All 30 enrolled individuals completed sample collection and clinical evaluation; therefore, no attrition or dropout occurred during the study. Stool samples were collected in sterile polypropylene containers and transferred to the laboratory within 10–15 min of collection. Samples were immediately frozen at −80 °C without preservative solutions and stored until DNA extraction.

### 2.2 Extraction of DNA from the stool samples

All DNA extractions were performed in a class II biosafety cabinet. After thawing at room temperature, an aliquot (0.2 g) of each sample was transferred into a sterile tube with 0.5 mm glass beads. Mechanical disruption of the samples was performed at 5000 xrpm for 1 min by a MagNA Lyser (Roche, Basel, Switzerland). Then, DNA was extracted from the stool samples using a DNA Stool Mini Kit (Qiagen, Germantown, MD, United States), according to manufacturer’s instructions. The quality and concentration of the DNA were determined with a NanoDrop spectrophotometer (Thermo Scientific Inc., Waltham, MA, United States) and a Qubit fluorometric assay (Life Technologies, Carlsbad, CA, United States). All DNA samples were stored at −80 °C until sequencing analysis.

### 2.3 16S rRNA sequencing

After isolation, the genomic DNA was amplified using bacterial 16S rRNA gene primers targeting the V3–V4 hypervariable regions with universal Eubacterial primers (forward; 5′-CCTACGGGNGGCWGCAG-3′) and (reverse; 5′-GACTACHVGGGTATCTAATCC-3′) (19). In the sequencing analysis, a 2-step PCR process was performed during library preparation. In these processes, 25 cycles of PCR were performed separately for each sample using the PrimeSTAR GXL DNA Polymerase (Takara, Kusatsu, Japan) enzyme. In the first PCR step, amplification was performed with an initial denaturation at 95 °C for 3 min, followed by 25 cycles consisting of denaturation at 95 °C for 30 s, annealing at 55 °C for 30 s, and extension at 72 °C for 30 s, with a final extension at 72 °C for 5 min. In the second PCR step, Nextera XT Index Primer 1 and Nextera XT Index Primer 2 sets (Illumina, San Diego, CA, United States) were used for the addition of Illumina index and adaptor sequences. In this PCR step, amplification was carried out with an initial denaturation at 95 °C for 3 min, followed by 8 cycles of denaturation at 95 °C for 30 s, annealing at 55 °C for 30 s, and extension at 72 °C for 30 s, with a final extension at 72 °C for 5 min. After both PCR processes, purification was performed with the Agencourt AMPure XP kit (Beckman Coulter, Brea, CA, United States). The concentration of the prepared libraries was measured using a Qubit fluorometer (Life Technologies, Carlsbad, CA, United States). Sequencing libraries were prepared using the NovaSeq S4 Reagent Kit (Illumina) and sequenced on the NovaSeq 6000 platform with paired-end reads, following the manufacturer’s guidelines.

### 2.4 Bioinformatic data processing

After sequencing, the quality of the raw reads was assessed using FASTQC to evaluate base quality, GC content, sequence length distribution, and potential adapter contamination. Low-quality bases, adapter sequences, and chimeric reads were removed using Trimmomatic v0.39 (20). Quality-filtered reads were then processed for taxonomic assignment. High-quality sequences were aligned to the RefSeq reference database (release February 2022) using the Kraken2 algorithm (21) for taxonomic classification. The resulting operational taxonomic units (OTUs) were identified at a 97% sequence similarity threshold. After quality filtering, sequencing depth was assessed across samples and found to be sufficient for downstream analyses. To account for differences in read depth, data were normalized prior to downstream analyses, and rarefaction was applied for alpha and beta diversity calculations, while relative abundance–based normalization was used for taxonomic comparison. Microbial diversity metrics were calculated using the vegan R package (22). Alpha diversity indices (Shannon, Simpson, and Inverse Simpson) were computed for all samples. Data visualization and statistical analyses were performed using R scripts and GraphPad Prism v9.0 (GraphPad Software, San Diego, United States).

### 2.5 CD4/CD8 ratio and percent activation of T cells

For each patient, whole blood was collected into EDTA-containing tubes for flow cytometric analysis of CD4 and CD8 cells activation. Red blood cells were lysed using a commercial lysis solution, and lymphocytes were fixed according to the manufacturer’s protocol. Absolute CD4 and CD8 cell quantification was carried out with human CD3, CD4, CD38, and HLA-DR antibodies for 10 min using Sysmex validated kits. Flow cytometric analysis was performed using a Sysmex XF-1600 cytometer (Sysmex Corporation, Kobe, Japan) with VenturiOne and Sysmex XF software. CD4/CD8 ratio was calculated from the respective absolute counts. Associations between CD4^+^ T-cell counts and gut microbiota composition were assessed using CD4^+^ measurements obtained from blood samples collected contemporaneously with stool sampling.

### 2.6 Statistical analysis

The data were analyzed using the IBM SPSS Statistics Standard Concurrent User V 26 software package (IBM Corp., Armonk, New York, United States). For graphical multivariate data visualizations and advanced statistical graph analyses, the JMP® Pro Version 17.0.0 software (SAS Institute Inc., Cary, NC, United States) was used. Descriptive data were expressed as mean ± standard deviation (SD) for normally distributed variables and as median (min–max) for non-normally distributed variables. The Shapiro–Wilk test was used to assess normality, and the Levene test was used to evaluate homogeneity of variances. When the assumptions for parametric tests were met, the One-Way Analysis of Variance (ANOVA) followed by Bonferroni *post-hoc* correction. For non-parametric data, the Kruskal–Wallis test was applied, with Dunn–Bonferroni adjustment for multiple comparisons. Pearson’s Chi-square test was used for categorical variables. A *p*-value of <0.05 was considered statistically significant. Multiple testing corrections were applied when necessary to minimize type I error. Alpha diversity indices (Shannon, Simpson, and Inverse Simpson) and beta diversity matrices were analyzed using the vegan R package (22) to evaluate within-group and between-group microbial diversity.

The study protocol was approved by the Local Ethics Committee (Approval No. 0283, dated June 2, 2022).

## 3 Results

### 3.1 Clinical characteristics of study cohort

Among the 30 HIV-positive patients, 27 were male, with a mean age of 43.9 years. The study included 10 in ART-naive patients (Group 1), 7 receiving ART for <2 years (Group 2), and 13 patients receiving integrase inhibitor–based ART for 2–5 years (Group 3). Only two patients had previous raltegravir and elvitegravir treatment which was switched to new generation integrase inhibitors in group 3. There was no other previous switch in ART in groups 2 and 3. The mean body mass index (BMI) was 24.46 kg/m^2^ (range: 19–33). The mean infection duration was 109 days. Regarding sexual orientation, 16 patients identified as heterosexual, 8 as men who have sex with men (MSM), and 3 as bisexual, while 3 patients did not specify their sexual orientation. There was no HBV or HCV co-infections. The mean plasma HIV-1 RNA level in Group 1 was 439,168 copies/mL. Between ART treated groups in Group 2 (ART for <2 years), all patients, except one (226 copies/mL), had undetectable viral loads. The mean CD4^+^ T-cell counts in Groups 1, 2, and 3 were 441.44 ± 275.41, 567.00 ± 162.93, and 799.61 ± 444.50 cells/mm^3^, respectively. With increasing duration of infection and prolonged ART exposure, HIV RNA levels decreased, while CD4^+^ T-cell counts increased. All patients were adherent to treatment, and virological suppression was achieved. The clinical and demographic information of the patients are summarized in Table 1. We also observed that ART-naïve individuals were younger than those in the ART-treated groups. As age may influence microbiota composition, this difference is acknowledged as a potential confounder.

**Table 1:** Demographic and clinical characteristics of HIV-positive patients stratified by treatment group.

Examinations	Group 1	Group 2	Group 3	*p* value
Number of individuals, *n* (%)	10 (33.3)	7 (23.3)	13 (43.4)	-
Age (years, mean ± SD)	36.30 ± 9.90	48.57 ± 16.48	47.23 ± 8.38	**0.042**
Gender, *n* (%)				
Female	1 (10.0)	0 (0.0)	2 (15.4)	
Male	9 (90.0)	7 (100.0)	11 (84.6)	
BMI (kg/m^2^, mean ± SD)	23.57 ± 2.53	24.42 ± 3.77	25.16 ± 3.58	0.753
Transmission mode, *n* (%)				
Heterosexual	5 (50.0)	3 (42.9)	8 (61.5)	0.053
MSM	4 (40.0)	1 (14.3)	3 (23.1)	
Unknown	1 (10.0)	0 (0.0)	2 (15.4)	
Bisexual	0 (0.0)	3 (42.9)	0 (0.0)	
Sexual oriantation, *n* (%)				
Heterosexual	6 (60.0)	3 (42.9)	8 (61.5)	0.030
MSM	4 (40.0)	1 (14.3)	3 (23.1)	
Unknown	0 (0.0)	3 (42.9)	0 (0.0)	
Bisexual	0 (0.0)	0 (0.0)	2 (15.4)	
Comorbid diseases during follow up, *n* (%)				
Stroke	7 (70.0)	2 (28.6)	12 (92.3)	0.166
Hyperlipidemia	1 (10.0)	0 (0.0)	0 (0.0)	
Hypertension	1 (10.0)	0 (0.0)	0 (0.0)	
Metastatic Pulmonary Carsinom	1 (10.0)	0 (0.0)	0 (0.0)	
Benign Prostatic Hyperplasia	1 (10.0)	0 (0.0)	1 (7.7)	
Osteoporosis	1 (10.0)	1 (42.9)	0 (0.0)	
Multiple Sclerosis	0 (0.0)	1 (14.3)	0 (0.0)	
Coronary Artery Disease	0 (0.0)	1 (14.3)	0 (0.0)	
Other medication, *n* (%)				
Statins	6 (60.0)	3 (42.9)	12 (92.3)	0.107
Antihypertensive	3 (30.0)	2 (28.6)	3 (23.1)	
Acetylsalicylic acid	2 (20.0)	0 (0.0)	0 (0.0)	
Vitamin complex	1 (10.0)	1 (14.3)	0 (0.0)	
Alendronic acid	0 (0.0)	1 (14.3)	0 (0.0)	
Quetiapine	0 (0.0)	1 (14.3)	0 (0.0)	
Beta blocker	0 (0.0)	1 (14.3)	0 (0.0)	
Clopidogrel	0 (0.0)	1 (14.3)	0 (0.0)	
Anxiolytic and antidepressant	0 (0.0)	2 (28.6)	0 (0.0)	
CD4 (mean ± SD)	441.44 ± 275.41	567.00 ± 162.93	799.61 ± 444.50	**0.023**
CD4/CD8 (mean ± SD)	0.39 ± 0.24	0.92 ± 0.29	0.91 ± 0.42	**0.003**
HIV RNA (copies/mL, mean ± SD)	497500.6 ± 560277.5	32.38 ± 85.41	2.38 ± 8.59	**<0.001**
Duration of Infection (mean ± SD)	0.30 ± 0.67	66.0 ± 99.90	94.30 ± 50.935	**<0.001**

### 3.2 Diversity of fecal microbiome

In HIV-1 positive patients receiving integrase-based treatment, fecal microbial diversity within groups was compared by alpha diversity (Shannon, Simpson, and Inverse Simpson indices).

**Figure 1:**
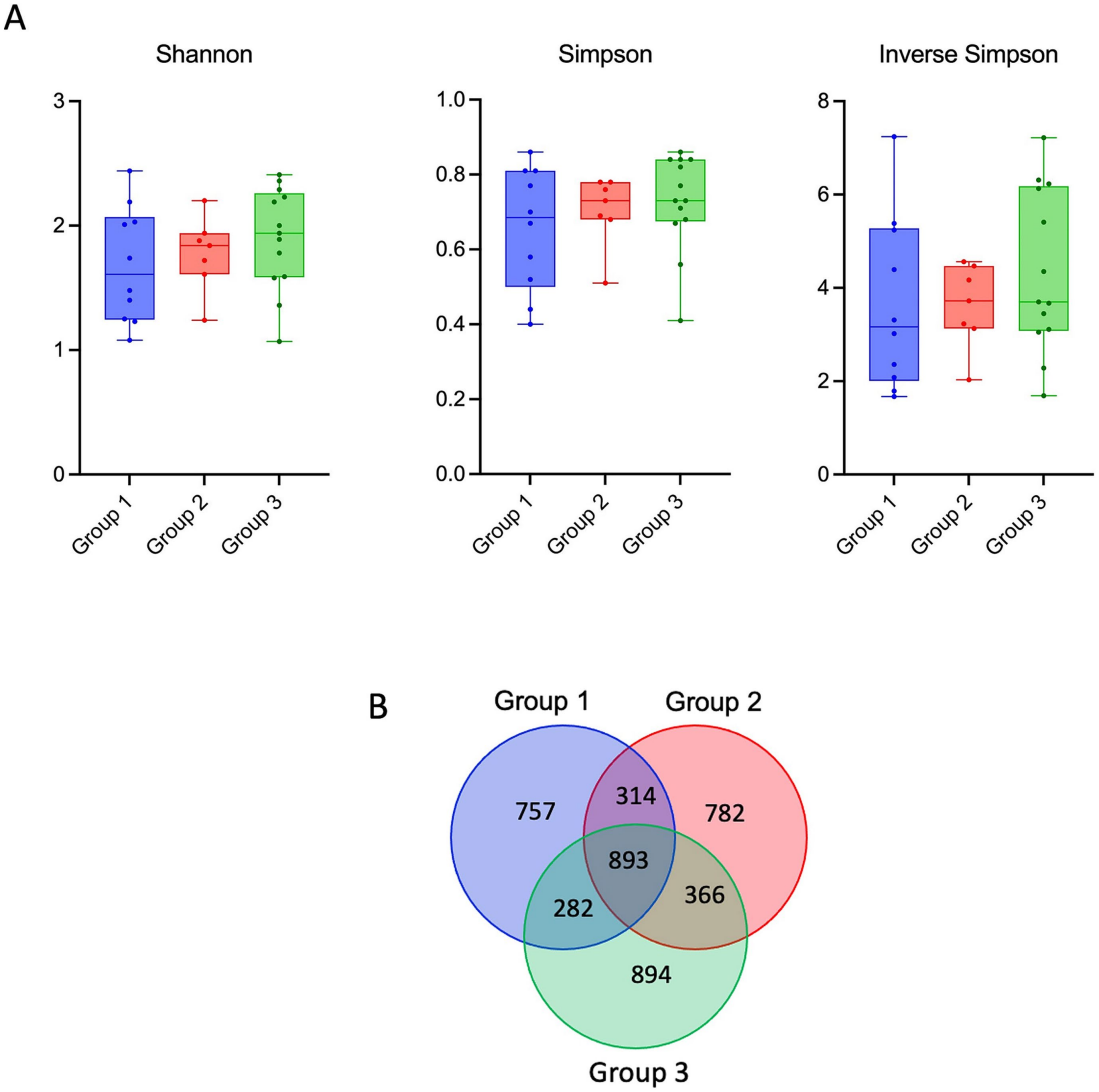
Diversity of groups. **(A)** Comparison of Shannon, Simpson, Inverse Simpson Index Values by Groups, **(B)** Venn diagram showing the number of shared and unique OTUs in fecal samples among the three patient groups based on 16S rRNA gene sequencing (97% similarity threshold). Patients were categorized into three groups according to ART exposure: Group 1, ART-naive patients; Group 2, patients on ART for <2 years; and Group 3, patients on integrase inhibitor–containing ART for 2–5 years.

No statistically significant difference in alpha diversity indices (Shannon, Simpson, Inverse Simpson) was observed among the three groups (*p* = 0.463). Nevertheless, both Shannon and Simpson indices showed a progressive, albeit modest, increase from ART-naïve to long-term INSTI-treated patients, in parallel with an increase in observed OTUs. These findings indicate a trend toward partial restoration of gut microbial diversity following integrase inhibitor–based ART rather than a definitive statistically significant effect (Figure 1A).

Based on 16S rRNA gene sequencing, an average of approximately 2,350 operational taxonomic units (OTUs) were identified across 30 fecal samples. Specifically, 2,246 species-level OTUs were found in Group 1, 2,355 OTUs in Group 2, and 2,435 OTUs in Group 3 based on a 97% similarity levels. The Venn diagram (Figure 1B) showes the overlap and unique OTUs among the groups.

### 3.3 Fecal microbiome composition among the groups

**Figure 2:**
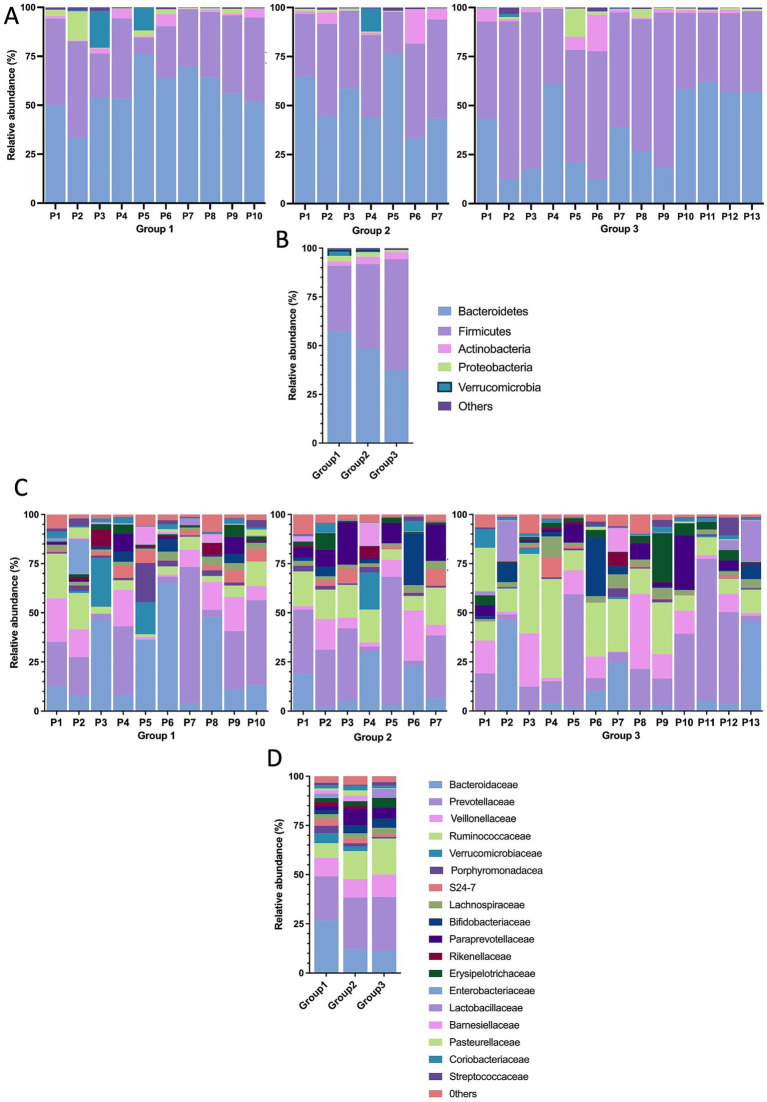
Structural composition of fecal microbiota. Relative abundance at the phylum level **(A,B)** and family level **(C,D)**.

At the phylum level, individuals were predominantly populated by Firmicutes and Bacteroides, with variable proportions of Actinobacteria. The relative abundance of Euryarchaeota (classified as others in the graph) was significantly higher in Group 2 than in Group 1 (*p* = 0.049). Similarly, Firmicutes significantly increased in Group 3 compared with Group 1 (*p* = 0.0029), whereas Bacteroidetes decreased (*p* = 0.0146). In contrast, Proteobacteria and Verrucomicrobia were more abundant in the ART-naïve group, and less Actinobacteria were observed (Figures 2A,B).

The abundance of Firmicutes was driven by the families of *Ruminococcaceae*, *Veillonellaceae*, *Lachnospiraceae*, and *Erysipelotrichaceae*. Interestingly, the abundance of Ruminococcaceae significantly increased with ART-treatment in both Groups 2 and 3 (*p* = 0,018, *p* = 0,013, respectively). The abundance of Bacteroidetes was driven by the families of *Prevotellaceae*, *Bacteroidaceae*, *Porphyromonadaceae*, *S24-7*, and *Barnesiellaceae*. Notably, Bacteroidaceae and Porphyromonadaceae were significantly lower abundance in both treatment groups than Group 1, with this reduction in Porphyromonadaceae being more pronounced in Group 3 (*p* = 0.039). On the other hand, Bifidobacteriaceae (Actinobacteria phylum) increased in both ART-treatment groups compared to Group 1, while Verrucomicrobiaceae, a member of the Verrucomicrobia phylum, decreased significantly, especially in Group 3. In addition, the abundance of Proteobacteria was due to the families of Pasteurellaceae, and Enterobacteriaceae. Although the abundance of Enterobacteriaceae decreased in both treatment groups, Pasteurellaceae exhibited a reduction in Group 3 and an increase in Group 2 (Figures 2C,D).

**Figure 3:**
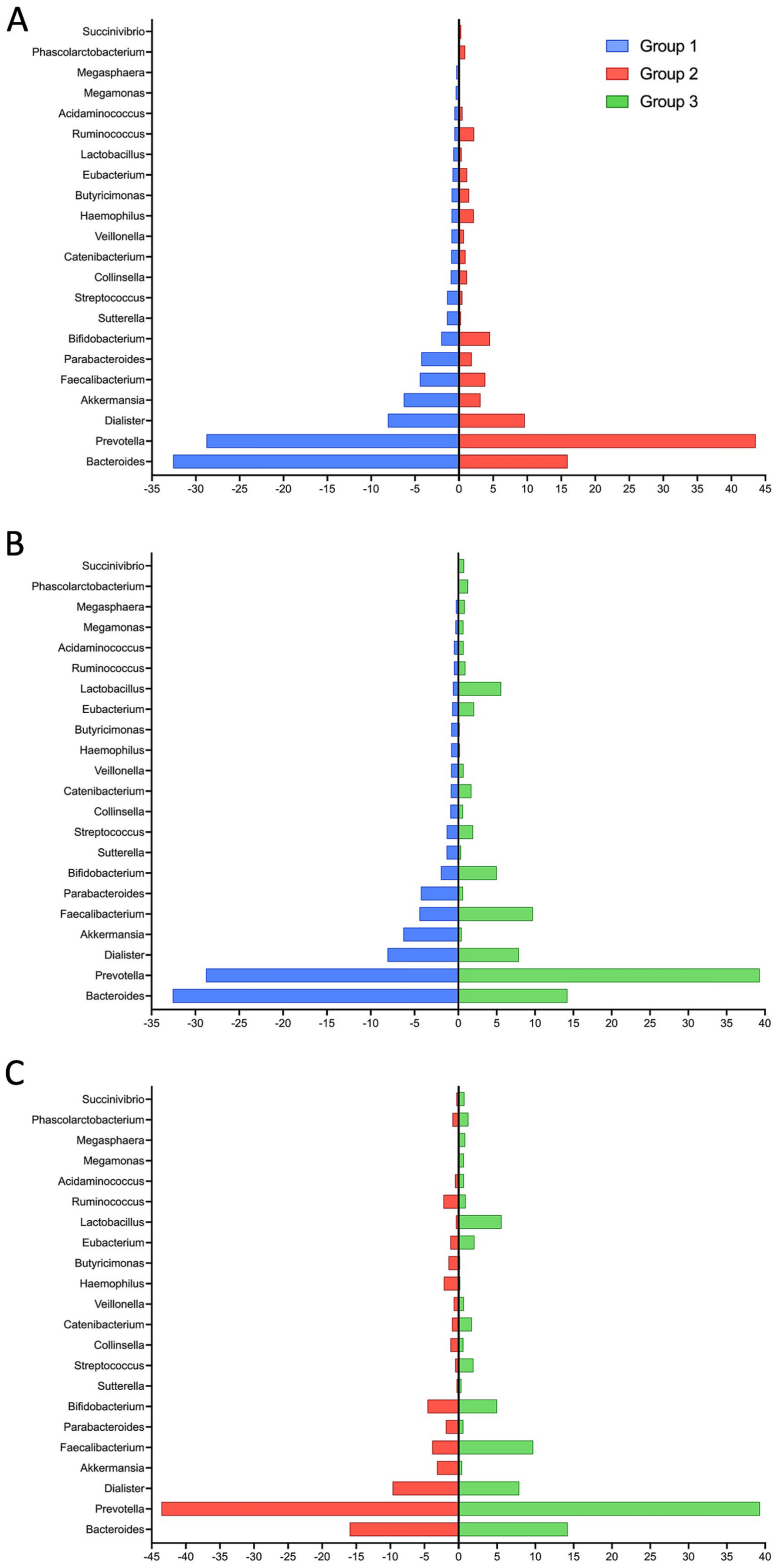
The highest and most diverse composition of the fecal microbiota at the genus level. Differences in relative abundance of genera were examined between groups. **(A)** Group 1 vs. Group 2, **(B)** Group 1 vs. Group 3, **(C)** Group 2 vs. Group 3.

At the genus level, taxonomic profiling revealed that *Bacteroides* and *Prevotella* belonging to phylum Bacteroidetes constituted the dominant genera across all groups. *Bacteroides* dominated in Group 1 but decreased significantly with ART-treatment. Conversely, the abundance of *Prevotella* exhibited a modest, yet consistent, increase in both treatment groups. *Faecalibacterium*, a beneficial short-chain fatty acid (SCFA) producer, decreased in Group 2 but increased significantly in Group 3 (*p* = 0.017), suggesting a restorative effect of long-term integrase inhibitor therapy (Figures 3A–C).

**Figure 4:**
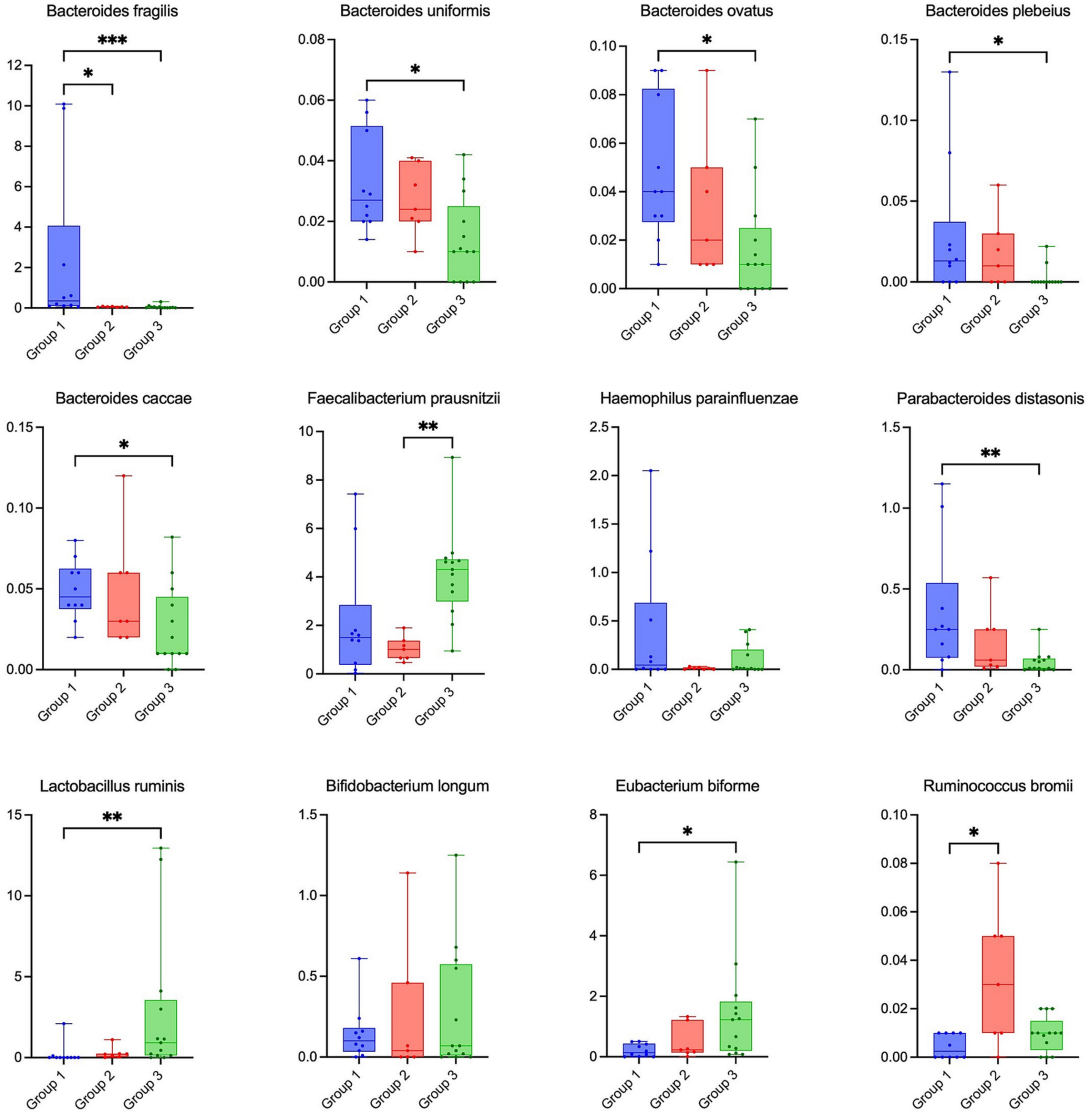
Alterations in key bacterial taxa at the species level across HIV treatment groups. Relative abundance of the species in HIV-infected patients groups.

Analysis at the species level revealed a significant depletion of several *Bacteroides* species in the treatment groups, most notably *B. fragilis, B. uniformis, B. ovatus, B. plebeius and B. caccae.* On the contrary, the abundance of *Faecalibacterium prausnitzii* displayed a marked disparity between the treatment groups, with a significant increase observed specifically in Group 3 (*p* = 0.0045). Furthermore, the intervention elicited significant enrichment of beneficial bacterial taxa, particularly within Group 3. This included pronounced increases in *Lactobacillus ruminis* (*p* = 0.0035), *Bifidobacterium longum,* and *Eubacterium biforme* (*p* = 0.032; Figure 4).

### 3.4 Association of fecal microbiome with CD4^+^ T cell count

**Figure 5:**
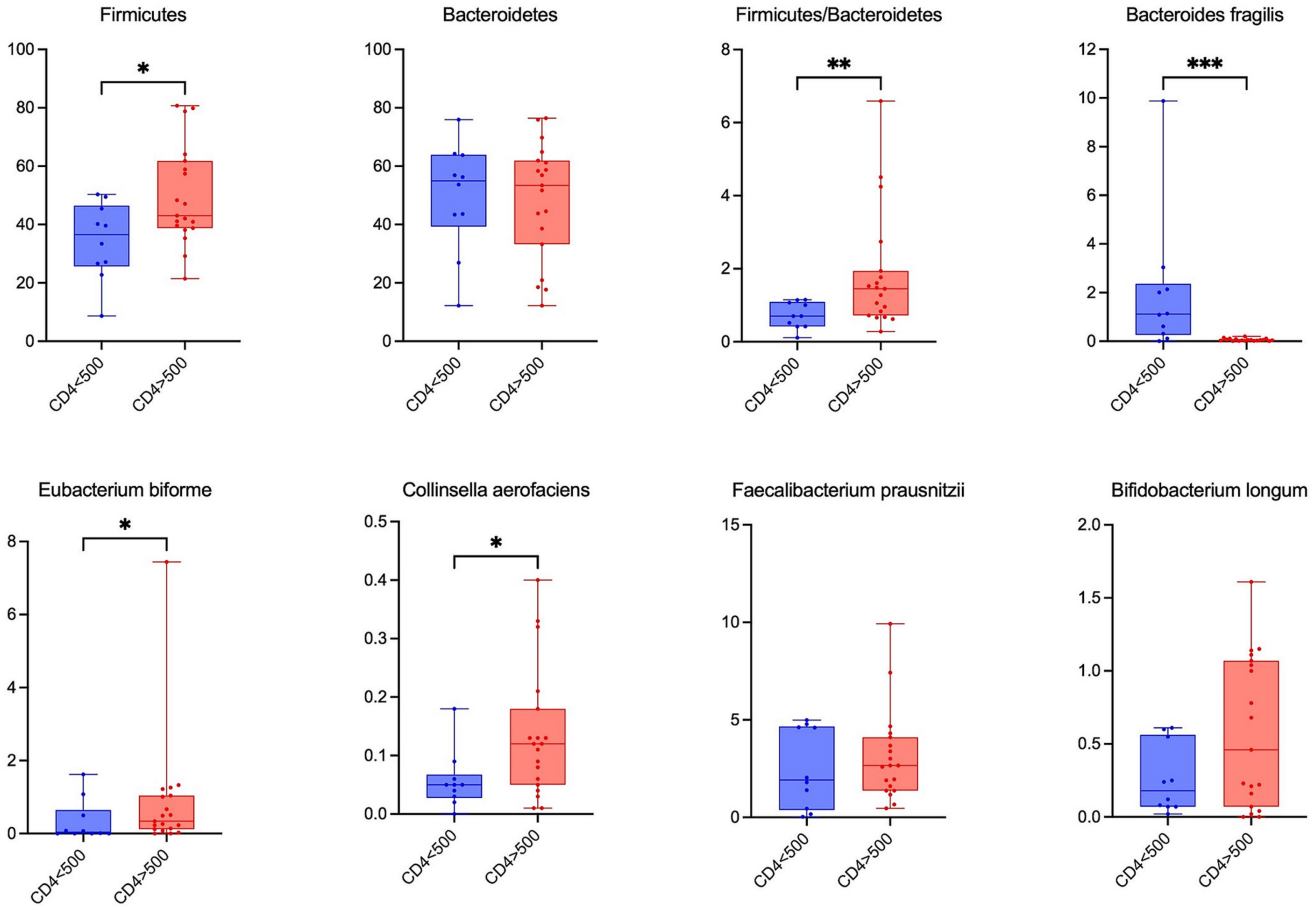
Association of fecal microbiome with CD4^+^ T cell count. Relative abundance of phyla and species in HIV-infected patient groups according to CD4^+^ T cell counts.

CD4 cell count is an indicator of immune function in patients living with HIV and one of the key determinants for the need of stage disease and inform clinical management. In the study, patients were stratified according to CD4^+^ T-cell count into low (<500 cells/mm^3^) and high (≥500 cells/mm^3^) groups (23). Comparative analysis of fecal microbiota composition revealed differences between HIV-infected individuals with low and high CD4^+^ T cell counts. At the phylum level, patients with low CD4^+^ T-cell counts exhibited a pronounced decrease in Firmicute*s* and a relative enrichment of Bacteroidetes and Proteobacteria, reflecting a dysbiotic microbial profile, compared to high CD4^+^ levels. Conversely, the Firmicutes/Bacteroidetes ratio increased in patients with higher CD4^+^ T cell counts (*p* = 0.007), indicating partial restoration of gut microbial balance during immune recovery. At the genus level, *F. prausnitzii, E. biforme, Collinsella aerofaciens,* and *B. longum*—all bacteria contributing to short-chain fatty acid production—were enriched in the group with higher CD4^+^ T cell counts. The former two are major butyrate producers, whereas the latter two contribute indirectly through cross-feeding interactions (Figure 5).

## 4 Discussion

### 4.1 Changes in diversity following integrase inhibitor–based ART

Integrase inhibitor–based ART (INSTI) regimens are currently recommended as the first-line therapy for HIV infection due to their strong antiviral efficacy, high genetic barrier, and minimal drug–drug interactions (24). The protease inhibitor-based regimen causes the greatest reduction in gut bacterial species, resulting in a loss of diversity (10).

The current study indicates that patients receiving INSTIs exhibited higher alpha diversity compared to ART-naïve patients group, although this difference did not reach statistical significance. This observation suggests a trend toward partial restoration of gut microbial diversity following long-term INSTI therapy, consistent with previous reports by Villoslada-Blanco et al. (17) and Villanueva-Millán et al. (10), who described similar trends after 1–13 years of INSTI treatment. Notably, in these studies, gut microbiota diversity and systemic inflammation markers in patients treated with INSTIs were found to be similar to those in HIV-negative individuals.

Additionally, some studies have concluded that protease-based ART regimens, in contrast to integrase inhibitors, cause more endothelial damage and microbial translocation, resulting in more inflammation (10, 15). This situation may be due to different mucosal penetration of ART regimens to gastrointestinal tissue. A study showed that it was shown that raltegravir, an integrase inhibitor, penetrates to gastrointestinal tract more rapidly (25). Furthermore, integrase inhibitors are also capable of rapid viral suppression, which can lead to rapid immune system reconstitution. The present study revealed that the modest increase in diversity indicates that ART alone cannot fully restore the reference microbiota state (ART naive), emphasizing the need for microbiota-targeted adjunct therapies. It is important to note that, to date, no universally accepted threshold defines what constitutes a ‘clinically meaningful’ change in alpha or beta diversity in people living with HIV receiving ART. Most studies interpret diversity metrics in a relative context and in combination with taxonomic shifts and markers of immune activation rather than as stand-alone clinical endpoints. In our cohort, the modest, non-significant increases in alpha diversity, together with the enrichment of SCFA-producing taxa and reductions in Proteobacteria and other pro-inflammatory bacteria, suggest a biologically plausible trend toward microbiota restoration. However, given the limited sample size and cross-sectional design, these findings should be considered exploratory, and we refrain from inferring a definitive clinically significant effect. Larger longitudinal studies that link microbiome dynamics with clinical outcomes are needed to establish the clinical relevance of such diversity changes.

Age is known to affect gut microbiota diversity and taxonomic composition, typically leading to reduced diversity and increased Proteobacteria in older adults. Although INSTI-treated groups in our cohort were older, they exhibited increased diversity and enrichment of SCFA-producing taxa patterns opposite to those expected with aging alone. Therefore, age is unlikely to fully account for the observed microbial differences. Nonetheless, given the modest sample size, age-related effects cannot be entirely excluded and should be examined in larger, age-matched cohorts.

### 4.2 Taxonomic shifts among study groups

At the phylum level, the gut microbiota in all groups was dominated by Firmicutes and Bacteroidetes, with Actinobacteria present at variable proportions, consistent with earlier studies (8, 10, 17). ART-naïve patients in this study displayed a reduction in Firmicutes and enrichments of Bacteroidetes, indicating dysbiosis. Following INSTI-based therapy, Firmicutes increased while Bacteroidetes decreased, reflecting a microbiota composition closer to healthy controls (17, 26). Similarly, Hanttu et al. (27) reported reduced Bacteroides after switching from NNRTI- to INSTI-based regimens. The Bacteroidetes phylum has been associated with Th17-mediated mucosal inflammation, whereas INSTI regimens may counteract this effect.

Proteobacteria and Verrucomicrobia were more abundant in ART-naïve patients, while Actinobacteria were depleted—findings consistent with Villoslada-Blanco et al. (17). Some discrepancies in Verrucomicrobia abundance among studies may reflect differences in diet, geography, or comorbidities. Similarly, some studies also found a relative abundance of Proteobacteria in patients receiving all forms of ART for at least 1 year (10, 16). However, other studies found no increase in the Proteobacteria population, which may be due to different types of samples (rectal biopsies, stool etc.) (28).

Consistent with previous findings, enrichment of Ruminococcaceae, Lachnospiraceae, Bifidobacteriaceae, and Veillonellaceae was observed at the family level—taxa known to produce SCFAs that are important for intestinal homeostasis in the population receving integrase inhibitor (12). Similar restoration of these SCFA-producing families after long-term ART has also been reported by Villanueva-Millán et al. (10). Notably, Bacteroidaceae, Porphyromonadaceae, and Verrucomicrobiaceae showed significantly lower abundance in both treatment groups, with this reduction in Porphyromonadaceae and Verrucomicrobiaceae being more pronounced in Group 3, similar to Villoslado-Blanco et al., (17). With the Proteobacteria phylum, the abundance of Enterobacteriaceae decreased in both treatment groups, whereas Pasteurellaceae showed a slight increase in Group 2 and a reduction in Group 3, in this study. Although some studies (10, 15) reported elevated Proteobacteria levels after long-term ART, others found no such increase (28), possibly due to differences in sample type, study population, or treatment duration.

At the genus level, Bacteroides and Prevotella (phylum Bacteroidetes) were the most abundant genera across all groups. Bacteroides predominated in Group 1 but decreased significantly following ART, particularly *Bacteroides fragilis*, which was markedly reduced in both treatment groups. Conversely, Prevotella showed a slight increase after therapy, consistent with prior reports (10, 15). These compositional shifts may reflect alterations in carbohydrate metabolism and partial mucosal adaptation associated with long-term ART exposure (10, 29–31).

The genus Faecalibacterium, particularly *F. prausnitzii*, decreased in Group 2 but increased significantly in Group 3, indicating a duration-dependent recovery under INSTI. F. prausnitzii is a key butyrate-producing commensal with strong anti-inflammatory activity, and its depletion has been linked to persistent gut inflammation (32, 33). This study revealed that the duration of INSTI treatment caused an increase in *F. prausnitzii*. Similarly, Narayanan et al. reported enrichment of Faecalibacterium in patients on long-term integrase-based regimens, supporting its potential role in mucosal immune restoration (34).

Notably, significant increases in *L. ruminis*, *E. biforme*, and *B. longum* were observed in individuals receiving long-term antiretroviral therapy, consistent with previous reports (9). These probiotic-associated species contribute to gut barrier integrity and immune modulation. Recent studies suggest that supplementation with these strains may help maintain microbial balance and alleviate gastrointestinal symptoms in HIV-infected individuals, although their direct impact on viral parameters remains limited (35, 36).

In interpreting these findings, it is also important to consider methodological aspects of microbiome profiling. Although 16S rRNA sequencing is a robust and widely accepted method for microbiota analysis, its limited taxonomic resolution may restrict the detection of subtle compositional or functional changes associated with ART exposure.

### 4.3 Association of fecal microbiome with CD4^+^ T cell

Numerous studies have indicated that the degree of immune preservation, as reflected by CD4^+^ T cell count, is a key determinant of intestinal microbial composition in HIV infection. When participants were stratified by CD4^+^ T cell counts, patients with low CD4^+^ levels showed a more pronounced dysbiosis characterized by decreased Firmicutes/Bacteroidetes ratio and increased Bacteriodes fragilis abundance compared to those with higher CD4^+^ counts. Spceially, in ART-treated subjects with immune reconstitution (CD4^+^ ≥ 500), *F. prausnitzii*, *E. biforme*, *Collinsella aerofaciens*, and *B. longum* were relatively enriched, indicating a positive association between These taxa are known producers or cross-feeders of SCFA, particularly butyrate, which plays a crucial role in maintaining intestinal barrier integrity and regulating immune homeostasis (32, 37). The enrichment of these butyrate-associated bacteria may therefore contribute to mucosal healing and improved CD4^+^ T cell recovery in ART treated individuals.

**Figure 6:**
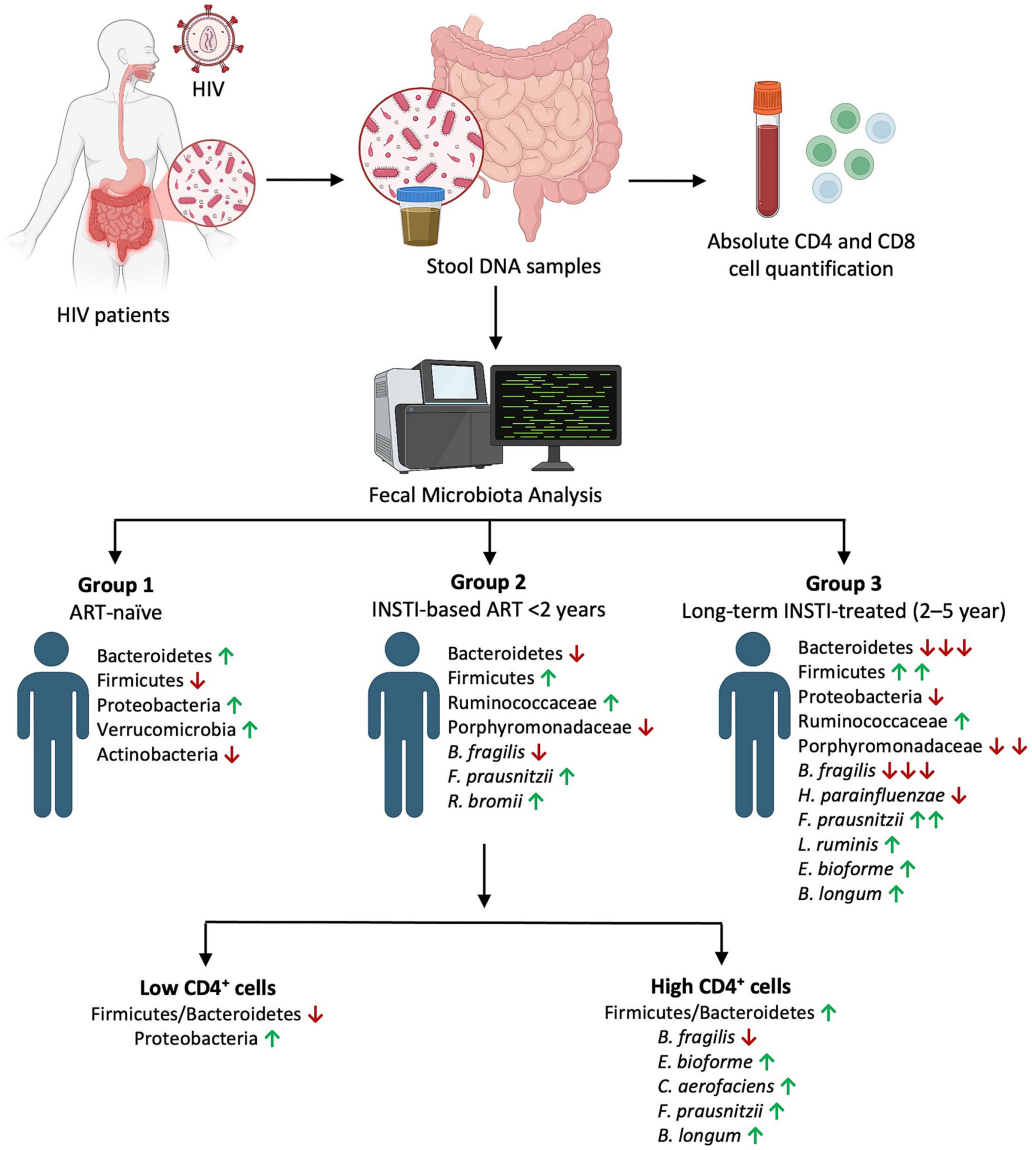
Changes in fecal bacterial taxa following ART treatment in HIV-infected patients and association of microbiome with CD4^+^ T cell.

Similarly, a study reported that *Bifidobacterium, Collinsella, Faecalibacterium, Oscillospira,* and *Roseburia* were enriched in HIV patients showing good immune reconstitution under ART (38). Interestingly, Lu and colleagues paradoxically found higher *F. prausnitzii* levels in treatment-resistant HIV cases (39), suggesting complex interactions between the gut microbiota and immune system reconstitution. Collectively, these findings suggest that both ART exposure and CD4^+^ T cell recovery contribute to a partial normalization of the gut microbiota in HIV infection (Figure 6).

### 4.4 Limitations

This study was limited by a small sample size, non-invasive mucosal sampling, and lack of control for diet and gender distribution. As patients were treated with different INSTI-based combinations, a degree of regimen-related heterogeneity may have influenced microbial patterns. However, given the shared pharmacological class and similar mucosal effects of INSTIs, the overall trends toward increased SCFA-producing taxa and reduced pro-inflammatory bacteria are biologically coherent. As all ART-experienced individuals in our cohort were receiving INSTI-based therapy, we were unable to compare microbiota profiles between INSTI regimens and other ART classes. While this limits the ability to attribute microbial changes specifically to INSTIs relative to PI- or NNRTI-based regimens, the within-class comparisons by treatment duration still provide insight into the trajectory of microbiota recovery under INSTI-based ART. Future studies including patients on alternative ART regimens are needed to determine whether these effects are unique to INSTIs. Although assessment of microbial translocation markers (e.g., LBP, LPS) and chronic inflammation markers (e.g., sCD14, sCD163, IL6) would have provided valuable insight into gut barrier integrity, these analyses could not be performed due to financial constraints, and this represents a limitation of the study. In addition the inclusion of an HIV-negative control group would have strengthened the study and provided a more appropriate baseline for comparison. Future studies including HIV- negative control groups would indeed be valuable to better contextualize our findings.

## 5 Conclusion

This study showed that gut microbiota composition in HIV-positive individuals differs according to treatment and immune recovery status. INSTI partially restored microbial diversity, increasing beneficial taxa, while reducing pro-inflammatory bacteria found in untreated patients. Conversely, ART-naïve individuals exhibited pronounced dysbiosis marked by reduced Firmicutes/Bacteroidetes ratio and enrichment of pro-inflammatory bacteria. These findings suggest that long-term INSTI-based regimens may help preserve fecal microbiota diversity, contributing to improved mucosal and immune function. The results highlight the potential role of microbiota-targeted strategies—such as dietary modulation or probiotic supplementation—as adjuncts to ART for improving intestinal and immune health in people living with HIV.

Future longitudinal studies integrating metagenomic and metabolomic analyses are needed to confirm these observations and clarify causal links between ART, microbiota restoration, and immune recovery.

## Data Availability

The original contributions presented in the study are publicly available. This data can be found in the NCBI BioProject database under accession number PRJNA1463720.
